# Inducible nitric oxide synthase and guinea-pig ileitis induced by adjuvant

**DOI:** 10.1155/S0962935195000044

**Published:** 1995-01

**Authors:** N. D. Seago, J. H. Thompson, X.-J. Zhang, S. Eloby-Childress, H. Sadowska-Krowicka, J. L. Rossi, M. G. Currie, P. T. Manning, D. A. Clark, M. J. S. Miller

**Affiliations:** 1 Department of Pediatrics Louisiana State University Medical Center New Orleans LA USA; 2 Monsanto/G. D. Searle St Louis MO USA

## Abstract

We sought to establish a model of inflammatory bowel disease by augmenting the activity of the local immune system with Freund's complete adjuvant, and to determine if inducible nitric oxide synthase (iNOS) expression and peroxynitrite formation accompanied the inflammatory condition. In anaesthetized guinea-pigs, a loop of distal ileum received intraluminal 50% ethanol followed by Freund's complete adjuvant. Control animals were sham operated. When the animals were killed 7 or 14 days later, loop lavage fluid was examined for nitrite and PGE_2_ levels; mucosal levels of granulocyte and macrophages were estimated by myeloperoxidase (MPO) and N-acetyl-D-glucosaminidase (NAG) activity, respectively. Cellular localization if iNOS and peroxynitrite formation were determined by immunohistochemistry with polyclonal antibodies directed against peptide epitopes of mouse iNOS and nitrotyrosine, respectfully. Adjuvant administration resulted in a persistent ileitis, featuring gut thickening, crypt hyperplasia, villus tip swelling and disruption, and cellular infiltration. Lavage levels of PGE_2_ and nitrite were markedly elevated by adjuvant treatment. Immunoreactive iNOS and nitrotyrosine bordered on detectability in normal animals but were markedly evident with adjuvant treatment at day 7 and particularly day 14. Immunohistochemistry suggested that enteric neurons and epithelia were major sites of iNOS activity and peroxynitrite formation. We conclude that local administration of adjuvant establishes a chronic ileitis. Inducible nitric oxide synthase may contribute to the inflammatory process.


Mediators of Inflammation 4, 19–24 (1995)

### Inducible nitric oxide synthase and guinea-pig ileitis induced by adjuvant

N. D. Seago,[1] J. H. Thompson,[1] X.-J. Zhang,[1] S. Eloby-Childress,[1] H. Sadowska-Krowicka,[1] J. L. Rossi,[1] M. G. Currie,[2] P. T. Manning,[2] D. A. Clark[1] and M. J. S. Miller[1,CA]

[1]Department of Pediatrics, Louisiana State University Medical Center, New Orleans, LA, USA and [2]Monsanto/G. D. Searle, St Louis, MO, USA

[CA] Corresponding Author

**We sought to establish a model of inflammatory bowel disease by augmenting the activity of the local immune system with Freund's complete adjuvant, and to determine if inducible nitric oxide synthase (iNOS) expression and peroxynitrite formation accompanied the inflammatory condition. In anaesthetized guinea-pigs, a loop of distal ileum received intraluminal 50% ethanol followed by Freund's complete adjuvant. Control animals were sham operated. When the animals were killed 7 or 14 days later, loop lavage fluid was examined for nitrite and $PGE_2$ levels; mucosal levels of granulocyte and macrophages were estimated by myeloperoxidase (MPO) and *N*-acetyl-D-glucosaminidase (NAG) activity, respectively. Cellular localization if iNOS and peroxynitrite formation were determined by immunohistochemistry with polyclonal antibodies directed against peptide epitopes of mouse iNOS and nitrotyrosine, respectfully. Adjuvant administration resulted in a persistent ileitis, featuring gut thickening, crypt hyperplasia, villus tip swelling and disruption, and cellular infiltration. Lavage levels of $PGE_2$ and nitrite were markedly elevated by adjuvant treatment. Immunoreactive iNOS and nitrotyrosine bordered on detectability in normal animals but were markedly evident with adjuvant treatment at day 7 and particularly day 14. Immunohistochemistry suggested that enteric neurons and epithelia were major sites of iNOS activity and peroxynitrite formation. We conclude that local administration of adjuvant establishes a chronic ileitis. Inducible nitric oxide synthase may contribute to the inflammatory process.**

**Key words:** Epithelium, Inflammation, Intestine, Leukocytes, Macrophage, Neuron, Nitric oxide, Peroxynitrite, Prostaglandin

### Introduction

Advances in our understanding of the pathophysiology of inflammatory bowel disease and potential therapeutic approaches to its management, require animal models that mimic the human condition. While the currently used models have proved to be useful and can predict the utility of current pharmacotherapies, it is still widely recognized that additional models are needed, particularly chronic models.[1] Hyperactivity of the gut-associated immune system is a hallmark of inflammatory bowel disease, although the triggers and factors maintaining this state are multiple and complex. Activation of immune defences with Freund's complete adjuvant (FCA), has been utilized in models of arthritis,[2] a common systemic complication associated with inflammatory bowel disease, but we are aware of only one report using this approach in gut inflammation.[3,4] Within this conceptual framework we attempted to establish a model of inflammatory bowel disease using FCA. The ileum and guinea-pig were chosen as the target organ and species, because of our previous experience with the trinitrobenzene sulphonic acid (TNBS) model.

It has recently been demonstrated that in contrast to reports demonstrating that nitric oxide is protective in acute models of gut injury,[5,6] nitric oxide synthase (NOS) inhibitors afford remarkable protective effects in chronic models of gut inflammation.[7] This apparent anomaly was termed the 'Jekyll and Hyde' role of nitric oxide in gut inflammation.[8] The reasons for this discrepancy appear to lie in the enzyme source of nitric oxide; injury appears to be associated with the inducible form of NOS (iNOS).[9,10] In human inflammatory bowel disease, nitric oxide production is exaggerated, with iNOS implicated as the source of NO.[11,12] In order to evaluate the potential role of NO in this new model of inflammatory bowel disease, we determined the magnitude of NO production, the cellular localization of iNOS expression and peroxynitrite formation by immunohistochemistry, the latter via the nitration of tyrosine residues.

### Materials and Methods

*Animals:* Fasted Hartley guinea-pigs of either sex (250–450 g) were anaesthetized by intramuscular

© 1995 Rapid Communications of Oxford Ltd injection of ketamine (40 mg/kg), xylazine (10 mg/kg) and atropine (0.2 mg/kg) supplemented with inhaled methoxyflurane as required. Under aseptic surgical conditions a midline laparotomy was performed. The distal ileum was isolated and gently flushed with intraluminal saline (2 ml) to remove residual enteric contents. Ethanol (50%, 0.5 ml) was injected transmurally into the lumen with a 26-gauge needle in order to disrupt the epithelial barrier. After 5 min the ethanol was gently manipulated distally and FCA (2 ml) was injected into the same site as ethanol. The FCA was held in place for 30 min in a 5 cm loop with a polyethylene tubing before the peritoneal cavity was closed by suturing layer by layer. The guinea-pig was transferred to a warm waterbed for post-operative recovery. Extensive pilot experiments in which FCA was evaluated by direct intramural injection and the ethanol/FCA volumes were varied, were performed before the current approach was determined to be the easiest and most reliable means of initiating gut inflammation. Sham animals received the same surgical procedure but saline, instead of ethanol and FCA, was injected into the intestinal lumen.

After 7 or 14 days the guinea-pigs were anaesthetized as described above. After flushing the ileal contents with warm saline, a 10 cm loop of ileum encompassing the region FCA administration, was made with silk ligatures. Saline (3 ml) was injected into the loop and after 30 min the loop was removed from the animal and the loop fluid volume recorded. The contents were centrifuged, divided into aliquots and frozen. Ileal thickness was determined from the ratio of weight to length; tissue water content and sections were collected for MPO and NAG (E.C. 3.2.1.30) content and fixation for routine histology and immunohistochemistry. Before the animals were killed by anaesthesia overdose, cardiac blood was drawn for quantification of peripheral leukocyte counts. All protocols were reviewed and approved by the institutional animal care and use committee, following NIH guidelines and in accordance with the Declaration of Helsinki.

*Ileal lavage analysis:* Loop fluid from ileal lavages were analysed for levels of reactive nitrogen intermediates (nitrite/nitrate) as an index of local production of NO, using a spectrophotometric technique as described previously.[7] Two methods were used: the Griess reaction or the fluorometric assay described by Misko and colleagues,[13] after conversion of nitrate to nitrite with bacterial-derived nitrate reductase (Sigma Chemical Co., St Louis, MO). While the two methods gave quantitatively different results the trends associated with treatments were comparable. Therefore, the Griess reaction results are shown and not the fluorometric assay results, because the Griess reaction is more widely used. In addition another inflammatory mediator was quantified in the loop fluid, prostaglandin $E_2$ ($PGE_2$) using an ELISA kit purchased from Cayman Chemical Company (Ann Arbor, MI). A Bio-Rad model 3550 plate reader was used for the Griess reaction and ELISA assays.

*Cellular infiltration:* Ileal MPO content was used as an index of tissue granulocyte content as described previously.[7,14] Briefly, ileal mucosal scrapings (100–250 mg) were finely minced at 4°C, homogenized with a Brinkman polytron for 20 s in 50 mM hexadecyltrimethylammonium bromide to inhibit the pseudoperoxidase activity of haemoglobin. The homogenate was centrifuged at 20 000 × ***g*** for 20 min at 4°C and the pellet was then frozen and thawed. This cycle of homogenization, freezing and thawing was repeated twice. Finally, an aliquot (100 μl) of the supernatant was added to 2.9 ml of potassium phosphate buffer (50 mM, pH 6.0) containing *o*-dianisidine dihydrochloride (0.167 mg/ml) and hydrogen peroxide (0.0005%). A Beckman DU-64 spectrophotometer was used to measure the absorbance at 460 nm over 2 min. One unit of MPO activity was set at that which degraded 1 μmol of $H_2O_2$ per min at 25°C.

Macrophage content in the ileum was estimated by NAG activity[15] in an analogous manner to the MPO assay for granulocytes. The NAG content was released from gut tissue by the same homogenizing and freeze–thawing procedure used for MPO. Assays were done in 96-well microtitre plates, 10 μl of sample or enzyme standard (Boehringer–Mannheim) were added together with 2.5 μl/well of substrate (*p*-nitrophenyl-2-acetamide-2-deoxy-D-gluco pyranoside, 2.24 mM Calbiochem, Ann Arbor, MI) and 100 μl/well of citrate buffer (50 mM, pH 4.5). Incubation was at room temperature for 60 min on a rotamixer (Barnstead Thermolyn type 51300). The reaction was stopped with a glycine buffer (100 μl, 200 mM). The yellow coloured product (*p*-nitrophenol) was read at 405 nm on a BioRad microtitre plate reader (model 3550).

Circulating leukocyte counts were performed from blood drawn by cardiac puncture. Blood (20 μl) was mixed with 180 μl of 3% acetic acid. White blood cells were counted using a Neubauer chamber under light microscopy.

*Morphometry:* Tissue was fixed in Bouin's solution and embedded in paraffin for staining with Masson's trichrome stain for collagen, or alternatively, with haematoxylin and eosin for routine histology. Immunohistochemical detection of iNOS was done in frozen tissue sections (5–10 μm) fixed with 1% formaldehyde. Vectastain Elite ABC kits from Vector Labs (Burlingame, CA) were used with a DAB substrate for identification and haematoxylin as a background stain. Antisera for nitrotyrosine was provided by Drs Joseph S. Beckman and Yao Zu Ye of the University of Alabama in Birmingham, USA. Tissue thickness was determined from the ratio of weight to length (mg/cm). Ileal water content was determined from the wet to dry weight ratios.

*Statistical analysis:* All data is expressed as the mean ± standard error. Groups were compared by analysis of variance and where valid, a Bonferroni-adjusted *t*-test was used to determine differences between individual groups. Kruskal–Wallis non-parametric tests were used for variations between more than two groups. Tests between groups used either Dunn's multiple comparison test or Tukey–Kramer multiple comparison test. The software package Instat was used to determine if tests were appropriate in addition to performing the analysis.

### Results

*Circulating and local leukocyte responses:* Ileal MPO content was markedly elevated at day 7 post-FCA administration, and then declined towards basal levels at day 14 (Table 1). NAG content remained above control values at both days ($p < 0.05$), but was markedly elevated at day 14. Total circulating leukocyte counts were elevated with FCA administration in a progressive manner over the 14 day study period (Table 1).

*Morphology and loop fluid analysis:* Loop lavage analysis revealed that FCA treated guinea-pigs had elevated local production of nitric oxide (as determined by nitrite) and $PGE_2$ at day 14 only (Table 2). Nitrite levels were determined by the Griess reaction or a fluorometric assay with comparable results ($r$ = 0.995). Ileal weight (mg/cm) was marginally increased in response to FCA treatment over the course of study (Table 2).

**Table 1.** Cellular responses in FCA-induced ileitis

| Group | Circulating leukocyte (cells/mm$^3$) | Ileal MPO activity (U/100 mg) | Ileal NAG activity (U/100 mg) |
|---|---|---|---|
| Control | 3109 ± 215 | 15.3 ± 12.2 | 91.0 ± 14.8 |
| FCA 7 days | 5384 ± 646** | 95.2 ± 36.6** | 112.4 ± 18.1 |
| FCA 14 days | 5954 ± 616** | 41.8 ± 8.3* | 409.4 ± 18.6**† |

Data expressed as mean ± S.E.M. *$p < 0.05$ *vs.* control. **$p < 0.01$ *vs.* control. †$p < 0.01$ *vs.* FCA days.

**Table 2.** Ileal responses to FCA administration

| Group | Ileal weight (mg/cm) | Lavage fluid nitrite (μM) | Lavage fluid $PGE_2$ (ng/ml) |
|---|---|---|---|
| Control | 72 ± 6 | 8.1 ± 1.2 | 0.47 ± 0.08 |
| FCA 7 days | 83 ± 3 | 48.3 ± 10.8** | 0.30 ± 0.06 |
| FCA 14 days | 85 ± 3* | 45.7 ± 0.8** | 1.50 ± 0.30*† |

Data expressed as mean ± S.E.M. Nitrite values determined by the Griess reaction. *$p < 0.05$ *vs.* control. † $p < 0.05$ *vs.* FCA 7 days. **$p < 0.01$ *vs.* control.

Histologically there was an increase in the extent of tissue injury from day 7 to 14. At both time-points there was a marked swelling of villus tips, with the epithelia becoming detached from the underlying basement membrane and rupturing (Fig. 1). Villus tips contained a large number of macrophages with vacuoles of cellular debris; macrophage accumulation on the basal surface of the detached epithelia was a common finding. The macrophages accumulating at the villus tips had a foamy appearance which presumably resulted from the ingestion of mineral oil in the adjuvant present in the mucosa.

Unlike the TNBS model in guinea pig ileum, FCA ileitis was associated with crypt abcesses (Fig. 1); these abcesses contained primarily macrophages and lymphocytes. In some sections it was evident that inflammatory cells extended from crypt abcesses between villi towards the lumen of the ileum. A marked hypercellularity of the crypts and absence of goblet cells was readily noted (Fig. 1). There was only minimal evidence of the inflammatory process in the muscularis; cellular infiltration appeared to be confined to the mucosa. Only a slight deposition of collagen in the submucosa was evident.

In normal guinea-pigs there was minimal positive staining for iNOS immunoreactivity. However, in the FCA-induced ileitis iNOS positive staining was evident in epithelia, particularly in terminal enterocytes and neurons in the submucosal and myenteric plexuses (Fig. 2). Nitrotyrosine immunoreactivity was located in the same sites as iNOS, being most evident in epithelia and myenteric neurons (Fig. 2). Incubation of the antibody with exogenous nitrotyrosine abolished the staining, indicating that positive staining was specific for nitrotyrosine present in the tissue.

### Discussion

Local administration of FCA initiates an inflammatory response in the guinea-pig ileum which is chronic in nature. Indices of gut injury and inflammation appear to be worse at day 14 compared with the appearance at day 7, with the exception of MPO activity. The diminished granulocyte content as determined by the MPO assay, probably reflects a change in the infiltrating cell type from granulocytes to macrophages and lymphocytes, as indicated in histological sections and by the NAG assay.

This approach to modelling inflammatory bowel disease, initially reported by Yamada *et al.* has great potential.[3,4] Yamada and colleagues reported that both complete and incomplete adjuvant could establish colitis in rats when administered directly into the bowel wall. The present approach using luminally applied adjuvant after epithelial exposure to a barrier breaker (ethanol) yielded similar results. Systemic levels of nitric oxide (nitrite/nitrate) were elevated in

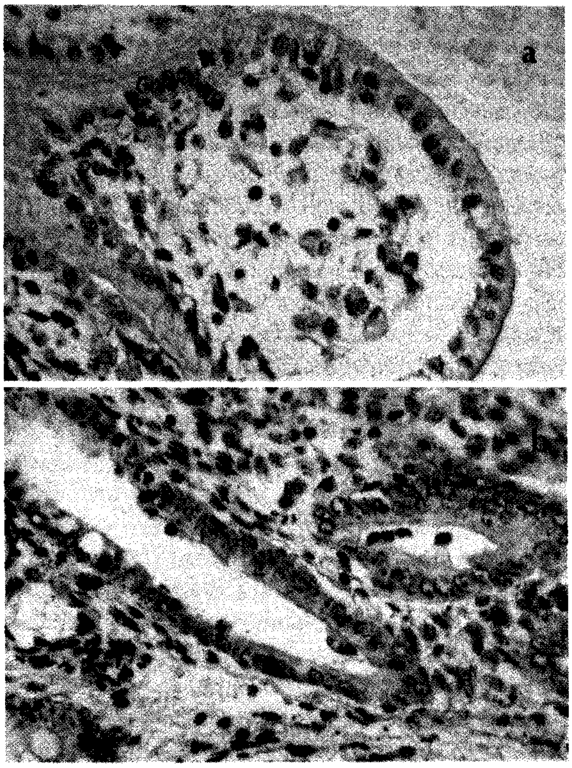

FIG. 1. Light photomicrographs of villus tips (Panel a) of adjuvant ileitis in guinea-pigs (original magnification x 200, haematoxylin and eosin staining). In Panel (a), the villus tip is swollen with detachment of the epithelia from the basement membrane. The lamina propria is rich in macrophages. In Panel (b), hyperplasia of the crypt epithelia is evident together with cellular infiltration (lymphocytes and macrophages) and a paucity of goblet cells.

the rat model of adjuvant colitis,[3,4] as has been demonstrated in adjuvant-induced models of arthritis.[16,17] Activation of local nitric oxide production was also observed in this study in guinea-pig ileitis.

However, does the current model accurately reflect events seen in human inflammatory bowel disease? Firstly, our model, established in the ileum, may be better suited as model of ulcerative colitis rather than Crohn's disease. Several observations led to this conclusion. The cellular infiltrate was largely confined to the mucosa and the crypt abcesses and epithelial disruption may better reflect ulcerative colitis than Crohn's disease. On the other hand the predominance of macrophages in the villus tips, particularly underlying detached epithelia at day 14, appears to be a unique feature and is not necessarily representative of any singular form of gut inflammation. It could serve as a useful model of macrophage-mediated injury. We have extensive experience with the TNBS model in the same species/site, and the FCA model appears to be less severe. Its distinguishing features are a relative lack of neutrophils but high numbers of macrophages and lymphocytes. Nevertheless, both models demonstrate an expression of iNOS. The advantage of the FCA model over the TNBS model, is that there is little damage or inflammation during the first week; rather, inflammation is established in the second week after a lag period. In

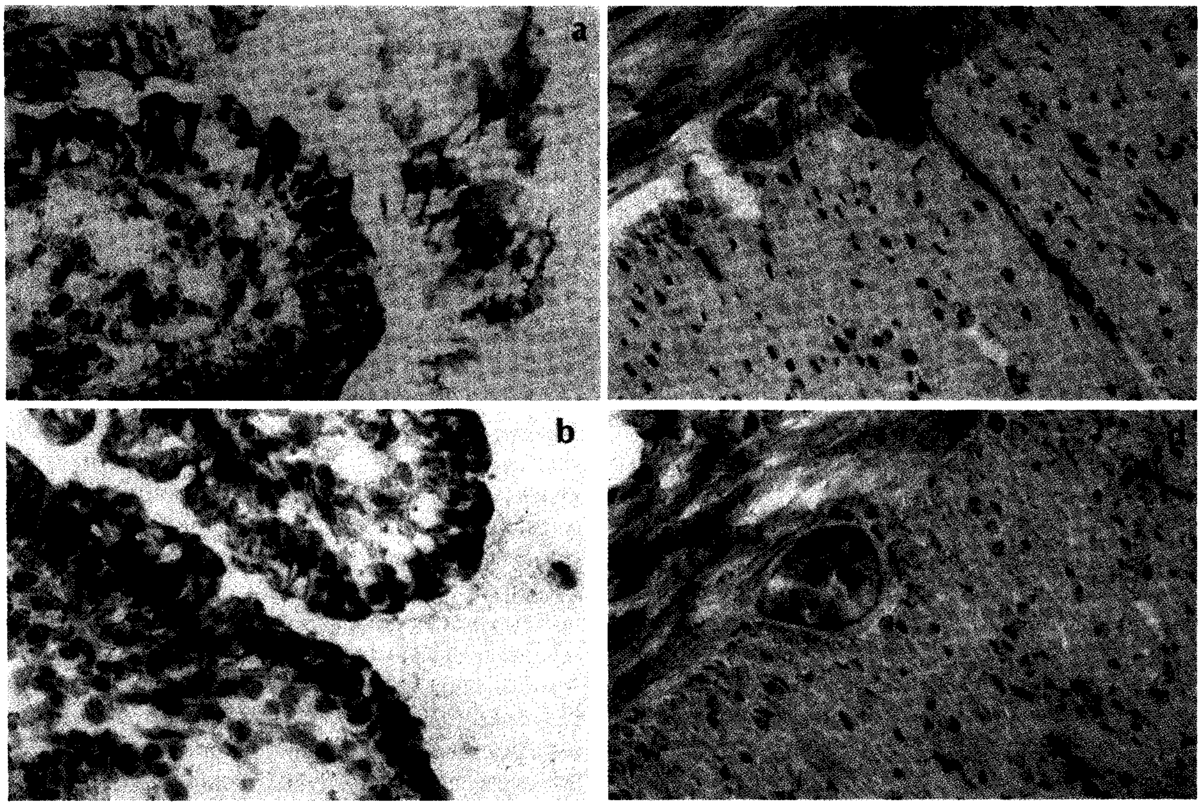

FIG. 2. Immunohistochemistry for iNOS and nitrotyrosine in terminal epithelia (Panels a and b respectively) and myenteric ganglia (Panels c and d respectively) of a guinea-pig 14 days after treatment with FCA. The DAB peroxidase method was used to visualize iNOS or nitrotyrosine; positive staining is indicated by a brown colour over the blue counterstaining of haematoxylin. Positive staining for both iNOS and nitrotyrosine was intense in epithelial cells and neurons of the myenteric plexus.

this regard it resembles the time course of adjuvant arthritis.

The FCA model that we have developed bears promise in that it is easy to initiate. Activation of the local immune system reflects the human condition where no single or primary pathogen is required, there are systemic consequences and the model is chronic. One may anticipate that reactivation of immunity by additional challenges, may prolong or reactivate this model of gut inflammation. Luminal application of FCA was chosen rather than the direct, subserosal approach of Yamada *et al.* because in our hands it is technically easier and more reliable.[3,4] It is particularly difficult to administer agents into the gut wall of small animals. Often the agent is administered into the lumen or on the serosal surface, where it could cause a peritonitis. With the use of ethanol as a barrier breaker, as previously described for the TNBS model, we avoid these complications but still manage to introduce the adjuvant into the mucosal tissue.[18,19] Injury induced by ethanol alone, 0.5 ml transmurally into the ileal lumen, is fully resolved by day 3,[19] and does not directly influence the interpretation of the data obtained at days 7 or 14. In pilot experiments to evaluate the model, no injury was found after a transmural injection of FCA alone.

An additional goal of the study was to determine if nitric oxide synthesized by the inducible form of NOS, contributed to tissue injury in this model. As previously noted in the TNSB model of ileitis,[7] FCA-induced inflammation is associated with an up-regulation of nitric oxide production. This appears to be the result of iNOS gene expression, with novel sites of induced NO production. The role of iNOS in this inflammatory response was evaluated with immunohistochemical techniques. It was observed that iNOS immunoreactivity was negligible or absent in normal controls but quite evident in FCA ileitis. Staining for iNOS was particularly intense in epithelial cells, predominantly mature enterocytes as there was little staining in crypt cells. Positive staining for iNOS was also observed in enteric neurons, both myenteric and submucosal. Again this was in response to the inflammatory state because iNOS staining was not apparent in normal controls. Expression of iNOS was associated with the formation of peroxynitrite, as indicated by nitrotyrosine immunoreactivity.[20] Nitric oxide itself cannot form nitrotyrosine, nitration of tyrosine involves $NO_2^+$, a degradation product of peroxynitrite ($ONOO^-$) or its protonated form which dominates at physiological pH. Peroxynitrite formation is normally associated with cell injury, suggesting that sites of iNOS expression are sites of cell injury or toxicity.

The expression of iNOS in epithelial cells may be interpreted as a defensive mechanism against invading microbes, with the production of a toxic chemical barrier. This response is persistent in the FCA model due to the continued presence of adjuvant (which contains mineral oil and dead mycobacteria) within the mucosa, possibly leading to enterocyte injury and increased cell turnover.

The presence of iNOS in enteric neurons is slightly more puzzling. Certainly dysmotility is a hallmark of gut inflammation but the nature or presence of altered gut motility is yet to be explored in this model. Neuronal cell injury due to excessive release of nitric oxide has been better explored in the central nervous system. As far as the authors are aware, those features described in the CNS, calcium overload in conjunction with NMDA receptor activation,[21,22] have not been evaluated in the gut.

In summary, local administration of FCA into the ileal mucosa establishes a chronic inflammation with features that suggest it may be a useful model for inflammatory bowel disease. As noted previously in the TNBS model of ileitis, FCA-induced inflammation initiates iNOS expression and excessive NO production. In the gut, the epithelia and enteric neurons are important sites of iNOS induction. Both the FCA model and role of iNOS and peroxynitrite in inflammatory bowel disease appear to be worthy of future investigation.

### References

1. Kim HS, Berstad A. Experimental colitis in animal models. *Scand J Gastroenterol* 1992; **27**: 529–537.
2. Taurog JD, Argentieri DC, McReynolds RA. Adjuvant arthritis. *Methods Enzymol* 1988; **162**: 339–355.
3. Grisham MB. Animal models of inflammatory bowel disease. *Curr Opin Gastroenterol* 1993; **9**: 524–533.
4. Yamada T, Zimmerman T, Specian RD, Grisham MB. Chronic granulomatous colitis induced by intramural injection of Freund's complete adjuvant. *Gastroenterology* 1993; **104**: A804.
5. Hutcheson IR, Whittle BJR, Boughton-Smith NK. Role of nitric oxide in maintaining vascular integrity in endotoxin-induced acute intestinal damage in the rat. *Br J Pharmacol* 1990; **101**: 815–820.
6. Caplan MS, Hedlund E, Hill N, MacKendrick W. The role of endogenous nitric oxide and platelet-activating factor in hypoxia-induced intestinal injury in rats. *Gastroenterology* 1994; **106**: 346–352.
7. Miller MJS, Sadowska-Krowicka H, Chotinaruemol S, Kakkis JL, Clark DA. Amelioration of chronic ileitis by nitric oxide synthase inhibition. *J Pharmacol Exp Therap* 1993; **264**: 11–16.
8. Miller MJS, Chotinaruemol S, Sadowska-Krowicka H, *et al.* Nitric oxide: the Jekyll and Hyde of gut inflammation. *Agents Actions* 1993; **39**: C180–C182.
9. Miller MJS, Clark DA. Nitric oxide synthase inhibition can initiate or prevent gut inflammation: role of enzyme source. *Agents Actions* 1994; (in press).
10. Thompson JH, Sadowska-Krowicka H, Rossi J, Clark DA, Miller MJS. Inducible nitric oxide synthase gene expression in guinea pig ileitis: a model of IBD prevented by aminoguanidine. *Gastroenterology* 1994; **106**: A782.
11. Boughton-Smith NK, Evans SM, Hawkey CJ, *et al.* Nitric oxide synthase activity in ulcerative colitis and Crohn's disease. *Lancet* 1993; **341**: 338–340.
12. Middleton SJ, Shorthouse M, Hunter JD. Increase nitric oxide synthesis in ulcerative colitis. *Lancet* 1993; **341**: 465–466.
13. Misko TP, Schilling RJ, Salvemini D, Moore WM, Currie MG. A fluorometric assay for the measurement of nitrite in biological samples. *Anal Biochem* 1993; **241**: 11–16.
14. Miller MJS, Zhang X-J, Sadowska-Krowicka H, *et al.* Nitric oxide release in response to gut injury. *Scand J Gastroenterol* 1993; **28**: 149–154.
15. Bailey PJ, Sturm A, Lopez-Ramos B. A biochemical study of the cotton pellet granuloma in the rat. *Biochem Pharmacol* 1982; **31**: 1213–1218.
16. Ialenti A, Moncada S, Di Rosa M. Modulation of adjuvant arthritis by endogeneous nitric oxide. *Br J Pharmacol* 1993; **110**: 701–706.
17. McCartney-Francis N, Allen JB, Mizel DE, *et al.* Suppression of arthritis by an inhibitor of nitric oxide synthase. *J Exp Med* 1993; **178**: 749–754.
18. Morris GP, Beck PL, Herridge MS, Depew WT, Szewczak MR, Wallace JL. Hapteninduced model of chronic inflammation and ulceration in the rat colon. *Gastroenterology* 1989; **96**: 795–803.
19. Miller MJS, Sadowska-Krowicka H, Jeng AY, *et al.* Substance P levels in experimental ileitis in guinea pigs: effects of misoprostol. *Am J Physiol* 1993; **265**: G321–G330.
20. Beckman JS, Ye YZ, Anderson PG, *et al.* Extensive nitration of protein tyrosines in human atherosclerosis detected by immunohistochemistry. *J Biol Chem* 1994; **375**: 81–88.
21. Manzoni O, Prezeau L, Marin P, Deshager S, Bockaert J, Fagni L. Nitric oxide-induced blockade of NMDA receptors. *Neuron* 1992; **8**: 653–662.
22. Boje KM, Arora PK. Microglial-produced nitric oxide and reactive nitrogen oxides mediate neuronal cell death. *Brain Res* 1992; **587**: 250–256.

ACKNOWLEDGEMENT. This work was funded by a grant from the Crohn's and Colitis Foundation of America, Inc.

**Received 9 August 1994;**
**accepted in revised form 8 September 1994**
